# Swiss Community Pharmacies' on the Web and Pharmacists' Experiences with E-commerce: Longitudinal study and Internet-based questionnaire survey

**DOI:** 10.2196/jmir.6.1.e9

**Published:** 2004-03-03

**Authors:** Simon Zehnder, Rudolf Bruppacher, Hans Ruppanner, Kurt E Hersberger

**Affiliations:** ^1^Institute for Clinical PharmacyPharmaceutical Care Research GroupPharmacenter, BaselSwitzerland; ^2^Documed AGBaselSwitzerland

**Keywords:** Web site, community pharmacies, professional-patient relations, quality of health care, e-commerce, e-pharmacies, Switzerland

## Abstract

**Background:**

There are multiple ways in which community pharmacies can present themselves on the Internet, e.g., as a platform for drug information or as an advertising platform for their services.

**Objective:**

To estimate the number of Swiss community pharmacies on the Internet over the period of 32 months (2000-2003), to describe their current e-commerce services, and to explore the experiences and plans these pharmacies have with regard to their Internet presence.

**Methods:**

A longitudinal study was performed to determine the number of Swiss German pharmacies on the Internet by conducting Internet searches in 2000, 2001, and 2003. In April 2002, a cross-sectional Internet-based survey was administered to explore the pharmacies' experiences and plans regarding their Web sites.

**Results:**

As of April 2003, 373 (44%) of 852 community pharmacies from the German speaking part of Switzerland were on the Internet. One hundred eighty four listed an e-mail address and were asked to complete a questionnaire. Of the 107 pharmacies answering the survey questions (58% response rate): 46% had been on the Internet for 1 to 2 years; 33% of the Web sites are part of a pharmacy group's Web portal; 31% of the pharmacies plan to expand their Internet appearance in the future; 74% provide e-commerce services, with 81% of those pharmacies filling five or less orders per month; and 12% plan on expanding their e-commerce services in the future.

**Conclusions:**

The number of community pharmacies offering Internet services steadily increased over 32 months. Given the importance of the Internet as a tool for information, communication, and advertising for pharmacy products and services, it can be expected that the increase will continue. Pharmacy-group portals are important promoters of pharmacies on the Internet. For many community pharmacies, Internet portals that provide an Internet presence for the pharmacies and provide regularly-updated content (e.g., health news, tips, drug information) seem to be the most effective solutions. Even though 40% of the pharmacies already offer e-commerce services, these services are still of minor importance. For many pharmacists, the current legal regulations seem to be unclear. Most pharmacies want to maintain their Internet services.

## Introduction

Worldwide, more and more people are using the Internet. In 2002, 49% of the Swiss population was reported to be online [1]. A pharmacy-specific Internet site can serve as a "Welcome Wagon", providing information about the pharmacy's hours, staff, location, and services. It can also provide a channel for patients to request information or refills, for physicians to prescribe, for pharmacists to assist patients with drug-related problems or to place an order with the wholesaler [2]. The Internet enables a pharmacist to communicate with patients through the use of e-mail, chat rooms, forums, videoconferencing, and other current or emerging Web communication formats [2]. Web sites created by community pharmacies could provide a quality filtered and customized information portal through which patients can access accurate, reliable drug information [2]. The Internet is the ideal tool for people to receive personalized tailored information about their disease states and medicines in the comfort of their homes and it provides more anonymity than face-to-face interactions with health care professionals, which is particularly relevant when asking embarrassing questions [3].

Given the rapidly-growing number of Web sites offering drugs on the Internet, community pharmacists are not willing to leave this new trade channel to often unidentifiable and dubious suppliers. Studies have shown that the drugs offered over the Internet are sometimes of bad quality and expensive; the origin of the drugs is often untraceable; or counseling is often unsatisfactory [4- 6]. An early paper, for example, showed that some e-pharmacies deliver drugs even if there are obvious contraindications [6]. There may be a considerable risk to patients' health. On the other hand, another study suggested that, under certain circumstances ( i.e., when online services are appropriately monitored and the right drugs are chosen), online prescriptions may be no more "potentially dangerous" than, for example, self-medication with over-the-counter (OTC) drugs [7]. A number of countries have proposed or implemented regulations for Internet pharmacies, and guidelines have been prepared on the quality of drug information for the patient on the Internet [8]. Ensuring the quality of Web sites and safeguarding consumers are both complex issues [9].

In Switzerland, several ways of distributing drugs via the Internet have emerged during the last couple of years. The federal law on medicinal products and medical devices, *Heilmittelgesetz*(HMG), does not contain any special regulations regarding the Internet, but it generally prohibits mail-order trade in medicinal products [10]. The states of the Swiss confederation that have legal sovereignty in health care can issue an authorization for mail order trade, if the following conditions are fulfilled:

- the medical product (including OTC products) has been prescribed by a medical doctor
- no safety requirements stand against it
- appropriate consultation is guaranteed
- sufficient medical supervision of the effect of the medicinal product is guaranteed.

A special form of mail-order trade that does not require a special authorization is shipping drugs to regular community-pharmacy customers. A pre-existing personal relationship (face-to-face) between the pharmacy and the patient is required. Furthermore, the service is only allowed in justified cases, e.g., if it is impossible for the patient to pick up the drug personally in the pharmacy [11]. Another option for a retail pharmacy is to affiliate with an Internet pharmacy partner (e.g., Wellshop in Switzerland [12]). The Internet-affiliation partner does not itself ship products to the patient's home but provides the necessary infrastructure (e.g., health and drug related information, 24-hour call-center) [2]. The patient either orders the desired product to be picked up in a local pharmacy or authorizes the shipment to his or her home [12].

In the view of many experts, pharmacies that combine a traditional retail operation with Internet-based business-to-consumer (B2C), so-called "bricks and clicks" pharmacies, are poised to become the most successful type of pharmacy in the future [13].

This study determines the presence of Swiss German community pharmacies on the Internet from 2000 to 2003, with special focus on e-commerce. The study addressed these questions:

- How many Swiss community pharmacies have an Internet presence?
- What are the characteristics of the community pharmacy Web sites?
- How many community pharmacies offer e-commerce services?
- What are the pharmacists' experiences and plans regarding their Internet presence?

## Methods

### Swiss Community Pharmacy Presence on the Internet

To determine the number of Swiss German community pharmacy Web sites, an Internet search was conducted in August 2000, with follow-up searches in December 2001, and April 2003. The Internet searches were performed with different search engines using the search term *Apotheke* *(pharmacy)* and two Swiss pharmacy Internet-address directories, Apo-Net and Switch. Apo-Net features a database of pharmacies maintained by Galexis, the largest drug wholesaler in Switzerland. Switch is the offical Swiss agency for the registration of Internet addresses with the top-level-domain.ch. We used their database to search for domain names containing the word "Apotheke". We used mainly Swiss search engines, to restrict the search to Swiss pharmacies. The search engines and directories used are listed in Table 1.

All hits found in the two directories were explored by clicking on each link. In addition, to complete the list, the first 200 hits found with each search engine were explored by clicking on each link. If the link turned out to be a pharmacy, name and Internet address was entered into a database. In addition, in the 2003 search, each identified Web site was checked for the presence of e-commerce services.

**Table 1:** Number of links found in various directories and search engines

Directory / Search engine	Number of Links Found		
	2000	2001	2003
Pharmacy Internet-Address Directories			
http://www.apo-net.ch/	64	56	117
http://www.switch.ch/	232	306	356
Search engines			
http://www.sear.ch/	3745	11470	396095
http://www.search.ch/	4637	9000	13000
http://www.google.ch/	17300	20900	27200
http://www.alltheweb.ch/	5217	6267	89163

### Internet-Based Survey

An Internet-based questionnaire survey (conducted from June 2002 to July 2002) was developed to explore the pharmacists' experiences with and future plans for their Web sites. The questionnaire had 21 items and was tested in a pilot survey among 20 pharmacies. Among the 235 pharmacies that were on the Internet (according to the Internet search of 2001, 184 had provided an e-mail address. These were approached by sending an e-mail that stated the purpose of the survey and invited them to take part. The participants were given the option of completing the questionnaire online or printing the questionnaire-attached to the e-mail as a portable document format (PDF) file-and send it back by fax or by regular mail. As an incentive, the participants were promised a summary of the survey results.

Questions had preformulated responses (yes-no options, multiple-choice options) and free-text fields for adding comments. Topics addressed included: (1) general information, (2) experiences or plans for the future, (3) patients' feedback, and (4) e-commerce. The original Internet-based questionnaire is in the Appendix.

Pharmacists who did not return the questionnaire in time were sent up to 2 reminders. In the second reminder, the nonrespondents were asked to state the reason (e.g., no time or no interest in the topic) for their nonparticipation.

Data-quality assurance was conducted by randomly selecting 5% of all cases following data entry and cross-checking with the coding sheet. No miscoded data was found. No cases had to be deleted due to very unlikely or extreme values.

### Data Collection and Statistics

The data (for all 3 survey parts) were collected in an anonymous manner and transferred to an Access database. SPSS (SPSS Inc, Chicago/IL, USA) was used for statistical analysis. Chi-square tests were used to test the type of Internet presence (individual Web site / part of pharmacy group's portal) and the offering of e-commerce or the duration of the Internet sites' existence and the offering of e-commerce. The a priori level of significance for type I errors (alpha) was set at <.05.

## Results

### Swiss Community Pharmacy Presence on the Internet

**Figure 1:**
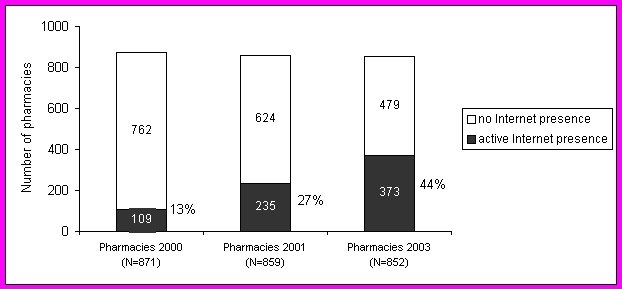
Number of community pharmacy Web sites: 2000, 2001, and 2003

In April 2003, 44% (373/852) of the Swiss German community pharmacies had an Internet presence (Figure 1). The denominator (total number of community pharmacies) was determined from the annual report published by the Swiss Pharmaceutical Society [14]. Among the 373 pharmacies with Internet presence, 150 (40%) offered e-commerce services, e.g., reservation of products via the Internet with self pick up in the pharmacy or delivery of products ordered via the Internet only to regular pharmacy customers.

The total number of pharmacies was obtained from the annual report published by the Swiss Pharmaceutical Society [14], while the number of pharmacies with active Internet presence was determined using Internet searches as described in this paper.

### Survey Results

Table 2-Table 4 show the results from the 2002 Internet-survey. Of the 184 pharmacies contacted via e-mail, 107 pharmacies answered (a 58% response rate).

**Table 2:** Basic characteristics of community pharmacy Web sites, as obtained through the 2002 Internet-survey.(n=107 respondents)

Characteristic	N	%
Duration of Internet presence < 1 year 1-2 years† 2-5 years† > 5 years Missing response	6 49 45 6 1	6 45 42 6 1
Type of Internet presence Individual Web site Part of a pharmacy group's Internet portal Missing response	71 35 1	66 33 1
Frequency of updates Weekly Monthly Yearly Irregularly Missing response	24 22 3 56 3	22 20 3 52 3
Communication with patients via e-mail Pharmacists who receive patients' e-mails Frequency Weekly Monthly Irregularly	98 8 22 68	92 8 22 70

^†^ Overlap of durations (1-2 years, 2-5 years) is an error from the questionnaire.

**Table 3:** Pharmacists' reasons for their Internet presence and future plans, as obtained through the 2002 Internet-survey.(n=107 respondents)

Variable	N	%
Reasons for Internet presence* Member of a pharmacy group's Internet portal Just to be part of the Internet The Web site provides additional value The Web site is a platform for independent information The Web site is an ideal source of advertising Other reasons	54 49 43 41 27 9	25 22 19 18 12 4
Future plans regarding the pharmacy Web site† Keep the Web site the same size Expand the Web site Downsize the Web site Discontinue the Web site	67 33 6 1	62 31 6 1

^*^ N = 223 answers; allowed multiple answers.

^†^ Based on 107 cases.

**Table 4:** Characteristics of the pharmacies offering e-commerce services, as obtained through the 2002 Internet-survey.(n=107 respondents)

Variable	N	%
E-commerce model* reservation of products via Internet with self pick up in the pharmacy affiliation with an Internet pharmacy partner delivery of products only to regular pharmacy customers delivery of products to all customers other models	55 37 34 17 4	37 25 23 12 3
Range of products† nonpharmaceutical products (e.g., cosmetics, dietary products) Over-the-counter (OTC) products products from in-house production Over-the-counter and prescription- products	70 45 36 29	39 25 20 16
Number of orders per month‡ up to 5 5-10 10-20 > 20	64 8 4 -	84 11 5 -
Concerns§ Regarding drug safety no concerns partial concerns large concerns missing response	38 51 10 8	35 47 9 7
Regarding safety of patient data: no concerns partial concerns large concerns missing response	61 37 1 8	56 34 1 7
Knowledge of the legal situation§ total knowledge partial knowledge no knowledge at all missing response	38 56 6 7	35 52 6 6
Future of e-commerce‡ keep e-commerce services expand e-commerce services reduce e-commerce services discontinue e-commerce services	59 9 5 1	80 12 7 1

^*^ N = 143 answers; allowed multiple answers.

^†^ N = 180 answers; allowed multiple answers.

^‡^ Addressed to the pharmacies offering E-commerce (N = 79); based on 74 to 76 cases due to missing responses. Overlap of durations (up to 5, 5-10, 10-20) is in the questionnaire.

^§^ Addressed to all the participants (N = 107)

### Basic Characteristics of the Community Pharmacy Web Sites

Basic characteristics of pharmacies online are shown in Table 2. Most pharmacies had offered Internet services for 1 to 2 years (45%) or for 2 to 5 years (42%). Sixty-two percent of the pharmacies had individual Web sites and 31% of the pharmacies had Web sites that were part of a pharmacy group's Web portal. Fifty-two percent of the pharmacies reported that their Web sites are updated irregularly and 42% reported weekly or monthly updates; 92% of the pharmacy owners said they had been contacted by patients via e-mail.

### Pharmacists' Reasons for Having an Internet Presence and Pharmacist's Future Plans

As illustrated in Table 3, 25% of the pharmacists stated they have an Internet presence because they are a member of a pharmacy group that includes all their members in an Internet portal and 22% stated they just want to be part of the Internet. Only 1 participating pharmacy plans to discontinue the Web site, while 31% of the pharmacies plan to expand their Internet presence.

As reasons for a planned expansion of the Internet site (n=31), the pharmacies stated that they want: to emphasize services in a more extensive way (34); to offer more-extensive counseling services via the Internet (e.g., provision of information regarding new drugs, health news, tips for consumers) (22); to offer e-commerce services (10); and to provide interactive communication channels (e.g., chats, forums, mailing lists) (3) (multiple answers were allowed).

As reasons for a planned downsizing (n=6) or discontinuation (n=1) of the Internet site, the pharmacies listed: financial aspects (6); expenditure of human labor (4); lack of customer feedback (4); data security (1); drug safety (1); and legal aspects (1) (multiple answers were allowed).

### E-commerce

According to our survey, e-commerce services were offered by 74% of the pharmacies. Three pharmacies that are not yet offering e-commerce said that it is out of the question for them to do so in the future. The other pharmacies that are not yet offering e-commerce services either want to monitor the market (36%), want to monitor the legal situation (21%), or are planning to introduce e-commerce services within the next 12 months (11%) or by a later point of time (21%). Table 4 illustrates the characteristics of the pharmacies offering e-commerce services.

Regarding the different ways of distributing drugs via the Internet, an Internet-based reservation system (37%) or affiliation with an Internet partner (25%) are the most popular e-commerce models. There was no significant association regarding either the type of Internet presence (individual Web site / part of pharmacy group's portal) and the offering of e-commerce (Χ ^2^_2_= 5.19. *P*> .05), or the duration of the Internet sites' existence and the offering of e-commerce (Χ ^2^_3_= 3.108, *P*> .05). Regarding the range of offered products, 16% offer pharmaceutical products, while nonpharmaceutical products (e.g., cosmetics, dietary products) are the most popular with 39% of pharmacies offering those. Most pharmacies (81%) fill only 5 or less orders per month. No pharmacy fills more than 20 orders per month. The majority of pharmacists expressed at least partial concerns regarding drug safety (56%), while a minority stated at least partial concerns with regard to the safety of patient data (35%) when providing e-commerce services. As reasons for the concerns regarding drug safety, the pharmacists stated among other things: "I am afraid of misuse of drugs"; "Is the Internet-customer trustworthy?"; "A face to face conversation is missing." Regarding concerns about the safety of patient data, the pharmacists stated: "Specific security systems (e.g., firewalls) are missing"; "Pharmacists don't have enough computer knowledge"; "There is a fear that submitted customer information could be seen by others." Thirty-eight percent of the participants stated they have total knowledge of the legal situatio n regarding e-commerce with drugs in Switzerland. Most pharmacies plan to keep (80%) or expand (12%) e-commerce services in the future, one pharmacy wants to withdraw from e-commerce services.

## Discussion

### Internet Presence

This study revealed that the number of pharmacies with an Internet presence doubled (109 in 2000; 235 in 2001) within the first 16 months and increased by another 59% (373 in 2003) within the second 16 months. A further increase can be expected, as there are still 479 community pharmacies (April 2003) that do not yet have an active presence on the Internet. In a recently-conducted survey (2001) with Swiss pharmacists, 41% stated they would like to be on the Internet with a Web site and 88% already had access to the Internet in the pharmacy [15]. As the results from this present study show, there are already more than those 41% with an Internet presence (44% in 2003). Regarding small- and medium-sized businesses in Switzerland, 33% were on the Internet in 2000 [16]. In 2001, 11% of the community pharmacies in Germany were on the Internet [17]. The importance of community pharmacy Web sites could gain importance in the future as, e.g., in the United States already 22% of the patients choose health care professionals based on information they find on the Internet [18].

One third of the pharmacies' Internet sites are part of a pharmacy-group's Internet portal. The pharmacy-groups' Internet portals (60% of the Swiss community pharmacies belong to chain or group organizations [19]) are a way for single pharmacies to become integrated into an Internet portal that is regularly updated by health care professionals and provides patients with up-to-date content (e.g., health news, tips, drug information).

### Plans/Reasons for Being on the Internet

As a recent Swiss patient survey revealed, 55% of patients do not feel the need for a community pharmacy Web site [20]. But this situation is likely to change. According a report by Striegler, community pharmacies without Web sites will lose a lot of customers [21]. More and more patients are likely to select pharmacies based on information they find on the Internet (e.g., on services pharmacies offer, such as diagnostics). Only a minority of the pharmacists plan on downsizing or giving up their Internet site in the future. Most of them seem to realize the importance of having a presence on the Internet, but only one-third plan to expand.

So far, only 19% of the pharmacists see their Web site as an additional value and only 18% as a platform for independent information, to become a drug-information center as proposed by experts in a recent survey [20,22]. One pharmacist's statement, that through the Internet "he wants to open the door to young customers," is a very important argument. Young people grow up with the Internet and its applications. A study by the Swiss Federal Office for Statistics described the typical Swiss Internet user as being young, male, and educated [1].

### E-commerce

In the summer of 2002, 74% of the Swiss pharmacies that were on the Internet provided e-commerce services. In Germany, this figure amounted to 32% in 2001 [17].

Seventy-two percent of the pharmacies that offer e-commerce services in Switzerland are affiliated with the Swiss Internet pharmacy partner Wellshop [12]. Of the 7500 monthly visitors to the Wellshop platform, only 1% used it to purchase OTC drugs online in 2001. Wellshop is currently mainly used as a drug-information platform [23]. A recent survey (2002) among Swiss pharmacy clients revealed that 2% of the Internet users have purchased drugs online [24]. Even though the number of online orders filled by the participating Swiss pharmacies is still very low, 80% would like to keep e-commerce services in the future and 12% would even like to expand their e-commerce services. The interest in e-commerce for drugs could increase in the future as 31% of the Swiss Internet users would be willing to order drugs online in the future [24].

Because relatively little information was known about the nonresponders, it could not be ruled out that pharmacies offering e-commerce services were overrepresented among the respondents. Regarding this possible bias, the pharmacy Web sites were checked for the presence of e-commerce services during the search of April 2003. The fact that in 2003 a total of 40% of the Web sites offered e-commerce services confirmed the assumption that the respondents of the 2002 survey might not be representative regarding the provision of e-commerce services (according to the 2002 survey, e-commerce services were offered by 74% of the pharmacies).

According to a British study, the 4 main groups buying pharmaceutical products online are: busy executives, mothers at home, the elderly or disabled, and those making embarrassing purchases [24]. The main reasons for buying drugs online are convenience and privacy [24,25].

Our finding that many pharmacists have only partial or no knowledge of the legal situation regarding e-commerce with drugs in Switzerland was confirmed by an investigation in 2003 [26]. It revealed that 34% of the pharmacies that are affiliated with Wellshop [12] do not require a prescription for ordering OTC-drugs even though, as stated in the federal law on medicinal products and medical devices (Heilmittelgesetz [10]), a prerequisite for mail order trade in medicinal products is a prescription by a medical doctor. It is important for community pharmacies to strictly respect the legal situation and apply the same level of quality they do when dispensing drugs in the traditional way. As explained in the "Introduction" for mail order, a prescription is needed for all kinds of drugs, including OTC drugs, which normally do not require a prescription.

### Limitations of the Survey

Relatively little information is known regarding the Internet-based survey nonrespondents. Only a minority of them stated the reason for not taking part in the survey. It cannot be ruled out that those who took part in the survey are more Internet savvy and are utilizing their Internet site in a more extensive way, e.g., regarding e-commerce. Furthermore, this study only measures the situation in Switzerland from 2000 to 2003. Considering the dynamic development in Internet presence and drug distribution over the Internet, this study would have to be repeated on a regular basis.

### Conclusions

The number of community pharmacies offering Internet services steadily increased over the 32 months covered by the survey. Because of the importance of this new medium as a tool for information, communication, and advertising for pharmacy products and services, the increase can be expected to continue. Pharmacy-group portals are important promoters of pharmacies on the Internet. For many community pharmacies, Internet portals that provide an Internet presence for the pharmacies and provide regularly-updated content seem to be the most effective solution. Even though 40% of the pharmacies already offer e-commerce services, it is still of minor importance. For many pharmacists, the current legal regulations seem to be unclear. Most pharmacies want to maintain their Internet services.

## Multimedia Appendix

### Original Internet-Based Questionnaire

Questionnaire "Pharmacies on the Internet: Survey concerning past experiences and future plans":

## Abbreviations

OTC: Over-the-counter (non-prescription)
