# Supplementary material for: Swiss Community Pharmacies' on the Web and Pharmacists' Experiences with E-commerce: Longitudinal study and Internet-based questionnaire survey
Source: J Med Internet Res. 2004 Mar 3;6(1):e9. doi: 10.2196/jmir.6.1.e9 (PMC1550588; doi:10.2196/jmir.6.1.e9)
Supplement: Supplementary file 1 [file jmir_v6i1e9_app1.html]

Fragebogen Apotheken im Internet


  

|  |  |
| --- | --- |
| **Fragebogen:** | **APOTHEKEN IM INTERNET** |
|  | Befragung zu bisherigen Erfahrungen und Zukunftsplänen |
|  |  |

**Dieser Fragebogen richtet sich an die 235 Deutschschweizer Apotheken
(Stand Dez. 2001), die bereits in irgendeiner Form im Internet, sei es durch
eine eigene Homepage oder eingebettet in ein Portal, vertreten sind. Da auch Ihr
Beitrag für mich sehr wertvoll ist, möchte ich Ihnen herzlich für Ihre Teilnahme
danken. Sie werden eine Zusammenfassung der Resultate erhalten. Ihre Antworten
werden in anonymer Form ausgewertet. Die Daten, die im Rahmen dieser Befragung
erhoben werden, werden ausschliesslich für wissenschaftliche Zwecke
verwendet.**

**Sie können
den Fragebogen bequem durch ancklicken der jeweiligen Antworten ausfüllen.
Fragen die mehrere Antworten erlauben, sind jeweils als solche gekennzeichnet.
Am Ende des Fragebogens gelangen die Resultate durch Drücken des "Senden" -
Knopfes an mich.**

**Bitte
senden Sie den ausgefüllten Fragebogen bis spätestens 1. September 2002
an mich zurück. Ich freue mich auf Ihre Antworten. Im Voraus bereits vielen Dank
für Ihre Mithilfe!**

**Falls
Sie noch Fragen oder Anregungen haben, können Sie mich jederzeit per E-Mail
kontaktieren.**

|  |  |  |  |
| --- | --- | --- | --- |
| **1.** | **ALLGEMEINES** | |  |
| **1.1** | **Seit wann sind Sie mit Ihrer Apotheke im Internet vertreten?** | |  |
|  | |  |  | | --- | --- | | seit <1 Jahr |  | | seit 1-2 Jahren |  | | seit 2-5 Jahren |  | | seit >5 Jahren |  | | |  |
|  |  | |  |
| **1.2** | **Welche Form hat Ihr Internetauftritt?** | |  |
|  | |  |  | | --- | --- | | Ihre Apotheke hat einen eigenständigen Internetauftritt (z.B. www.meineapotheke.ch) |  | | Ihr Internetauftritt ist eingebettet in ein Portal (z.B. unter www.apoguide.ch / www.conpharm.ch / www.feelgoods.com / www.toppharm.ch) |  | | Sie haben sowohl einen eigenen Internetauftritt als auch einen Internetauftritt der in ein Portal eingebetet ist. |  | | |  |
|  |  | |  |
| **1.3** | **Wie oft wird Ihre Internetseite aktualisiert?** | |  |
|  | |  |  | | --- | --- | | wöchentlich |  | | monatlich |  | | jährlich |  | | in unregelmässigen Abständen |  | | |  |
|  |  | |  |
|  |  | |  |
| **2.** | **ERFAHRUNGEN / ZUKUNFTSPLÄNE** | |  |
| **2.1** | **Wird Ihr Auftritt im Internet von Ihren Kunden / Patienten wahrgenommen?** | |  |
|  | Ja, ich habe bereits ein Echo von Kunden / Patienten bekommen  (z.B. via E-Mail, Bestellungen, mündliche Rückmeldungen):   |  |  | | --- | --- | | mehrmals wöchentlich |  | | mehrmals monatlich |  | | mehrmals im Jahr |  | | einzelne Male |  | | |  |
|  | Ich habe bisher noch keine Rückmeldungen von Kunden/Patienten bekommen. | |  |
|  |  | |  |
| **2.2** | **Wie sehen Ihre Zukunftspläne bezüglich Ihrem Internetauftritt aus?** | |  |
|  | |  |  | | --- | --- | | Ich möchte den Umfang des jetzigen Internetauftrittes beibehalten. | ***weiter bei 2.5*** | | Ich möchte den Internetauftritt ausbauen. | ***weiter bei 2.3*** | | Ich möchte den Internetauftritt reduzieren. | ***weiter bei 2.4*** | | Ich möchte den Internetauftritt aufgeben. | ***weiter bei 2.4*** | | |  |
|  |  | |  |
| **2.3** | **Gründe für einen möglichen Ausbau des Internetauftrittes:**  *(mehrere Antworten möglich)* | |  |
|  | |  |  | | --- | --- | | Verstärkt auf Beratung via Internet setzen (durch z.B. Informationen über Arzneimittel, allgemeine Gesundheitsthemen, News, Tipps) |  | | Den Benutzern die Möglichkeit bieten sich in Chats, Foren, Mailinglisten auszutauschen. |  | | Die Dienstleistungen der Apotheke stärker betonen. |  | | Ausbau in Richtung E-Commerce mit Arzneimitteln. |  | | |  |
|  | Andere Gründe: | |  |
|  | ***weiter bei 2.5*** | |  |
|  |  | |  |
| **2.4** | **Gründe für eine mögliche Reduktion/Aufgabe des Internetauftrittes:**  (mehrere Antworten möglich) | |  |
|  | |  |  |  |  |  |  | | --- | --- | --- | --- | --- | --- | | Aufwand zahlt sich nicht aus:  |  |  | | --- | --- | | finanzieller Aufwand: |  | | Arbeitsaufwand: |  | |  | | Ungenügende Resonanz seitens der Kunden. |  | | Bedenken wegen der Gewährleistung der Datensicherheit. |  | | Bedenken wegen der Gewährleistung der Arzneimittelsicherheit. |  | | Gesetzgebung des neuen Heilmittelgesetzes. |  | | |  |
|  | Andere Gründe: | |  |
|  |  | |  |
| **2.5** | **Haben Sie bereits Anfragen von Kunden/Patienten zur Beantwortung  von Fragen via E-Mail erhalten?** | |  |
|  | |  |  | | --- | --- | | ja |  | | nein |  | | |  |
|  | Falls Ja: Wie oft?   |  |  | | --- | --- | | mehrmals wöchentlich |  | | mehrmals monatlich |  | | mehrmals im Jahr |  | | einzelne Male |  | | |  |
|  |  | |  |
| **2.6** | **Sie sind mit Ihrer Apotheke im Internet vertreten weil:**  (*mehrere Antworten möglich*) | |  |
|  | Sie einfach dabei sein wollen. |  |  |
|  | Sie das Internet als ideales Werbemedium für Ihre Apotheke sehen. |  |  |
|  | Sie darin einen echten Mehrwert für Ihre Kunden / Patienten sehen. |  |  |
|  | Sie Ihren Kunden / Patienten eine Plattform mit unabhängigen, aufgearbeiteten Informationen bieten möchten. |  |  |
|  | Sie einer Apothekengruppierung angehören die einen gemeinsamen Internetauftritt hat. |  |  |
|  | Andere Gründe: | |  |
|  |  | |  |
|  |  | |  |
| **3.** | **E-COMMERCE / VERSANDHANDEL MIT ARZNEIMITTELN** | |  |
| **3.1** | **Bieten Sie über Ihre Internetseite die Möglichkeit an, Produkte  (z.B. Arzneimittel, Parapharmazie, Hausspezialitäten) zu bestellen  oder zu reservieren?** | |  |
|  | |  |  | | --- | --- | | ja | ***weiter bei 3.3*** | | nein | ***weiter bei 3.2*** | | |  |
|  |  | |  |
| **3.2** | **Falls Sie noch keine Bestell- / Reservationsmöglichkeit über  Ihre Internetseite anbieten: Wäre es für Sie in Zukunft denkbar, Ihre  Internetseite in Richtung Versandhandel auszubauen?** | |  |
|  | Ja, das könnte ich mir vorstellen:  |  |  | | --- | --- | | innerhalb der nächsten 6 Monate |  | | innerhalb der nächsten 12 Monate |  | | später |  | |  |  | | Nein, das käme für mich auf keinen Fall in Frage. | ***weiter bei 4***. | | Ich warte zurzeit noch ab und beobachte den Markt. |  | | Ich werde mich je nach rechtlicher Situation entscheiden. |  | | |  |
|  |  | |  |
| **3.3** | **Falls Sie bereits E-Commerce anbieten oder es in Zukunft anbieten  möchten: Welches Modell nutzen Sie / käme für Sie in Frage?**  (*mehrere Antworten möglich*) | |  |
|  | |  |  | | --- | --- | | Reservierung der Arzneimittel durch den Kunden über das Internet mit Selbstabholung durch den Kunden in der Apotheke. |  | | Versand von online bestellten Arzneimitteln nur an bekannte Kunden. |  | | Versand von online bestellten Arzneimitteln an alle. |  | | Beteiligung an einem bereits bestehenden E-Commerce-System (z.B. wellshop.ch) |  | | |  |
|  | Anderes: | |  |
|  |  | |  |
| **3.4** | **Welche Produkte bieten Sie an bzw. möchten Sie anbieten?** (*mehrere Antworten möglich*) | |  |
|  | |  |  | | --- | --- | | nur Produkte aus dem OTC-Bereich |  | | sowohl OTC als auch rezeptpflichtige Produkte |  | | Parapharmazie |  | | Hausspezialitäten |  | | |  |
|  | Anderes: | |  |
|  |  | |  |
| **3.5** | **Falls Sie bereits eine Bestell- / Reservationsmöglichkeit über Ihre Internetseite  anbieten: Wie viele Bestellungen / Reservationen führen Sie monatlich aus?** | |  |
|  | |  |  | | --- | --- | | bis 5 |  | | 5-10 |  | | 10-20 |  | | >20 |  | | |  |
|  |  | |  |
| **3.6** | **Sicherheit: Haben Sie Bedenken:** | |  |
|  | Bezüglich der Gewährleistung der **Arzneimittelsicherheit** bei der  Online-Bestellung / Reservation von Arzneimitteln über Ihre Internetseite? | |  |
|  | |  |  | | --- | --- | | nein, überhaupt keine |  | | zum Teil |  | | ja, grosse |  | | |  |
|  | Gründe für Bedenken : | |  |
|  |  | |  |
|  | Bezüglich der Gewährleistung der Sicherheit von **Patientendaten** auf  ihrem Computersystem? | |  |
|  | |  |  | | --- | --- | | nein, überhaupt keine |  | | zum Teil |  | | ja, grosse |  | | |  |
|  | Gründe für Bedenken : | |  |
|  |  | |  |
| **3.7** | **Kennen Sie die aktuelle Gesetzgebung hinsichtlich dem Versand  von Arzneimitteln über das Internet?** | |  |
|  | |  |  | | --- | --- | | ja, vollumfänglich |  | | teilweise |  | | nein, überhaupt nicht |  | | |  |
|  |  | |  |
| **3.8** | ***Falls Sie bereits E-Commerce anbieten*: Möchten Sie in Zukunft  die Bestell- / Reservationsmöglichkeit von Arzneimitteln über  Ihre Internetseite beibehalten?** | |  |
|  | |  |  | | --- | --- | | Ja, ich möchte eine Bestellmöglichkeit von Arzneimitteln über meine Internetseite im jetzigen Umfang auch in Zukunft beibehalten. |  | | Ich möchte den Versandhandel von Arzneimitteln über meine Internetseite in Zukunft weiter ausbauen. |  | | Ich möchte den Versandhandel von Arzneimitteln über meine Internetseite reduzieren. |  | | Ich möchte den Versandhandel von Arzneimitteln über meine Internetseite aufgeben. |  | | |  |
|  | Gründe für einen Ausbau des E-Commerce: | |  |
|  | Gründe für eine Reduktion / Aufgabe des E-Commerce: | |  |
|  |  | |  |
|  |  | |  |
| **4.** | **ANGABEN ZUR PERSON / APOTHEKE** | |  |
| **4.1** | Bitte geben Sie an, in welcher Position Sie sind und ob Sie für den Internetauftritt  verantwortlich sind.   |  |  |  | | --- | --- | --- | | **Position:** | | **Für den Internetauftritt verantwortlich:** | | Pharmaassistentin |  |  | | Pharmabetriebsassistentin |  |  | | angestellte Apothekerin / angestellter Apotheker |  |  | | GeschäftsführerIn / VerwalterIn / BesitzerIn |  |  | | |  |
|  |  | |  |
| **4.2** | **Lage** | |  |
|  | |  |  | | --- | --- | | Stadt (City, Passantenlage) |  | | Stadt (Quartierlage) |  | | Agglomeration |  | | Dorf / Land |  | | |  |
|  |  | |  |
| **4.3** | **Es überwiegt** | |  |
|  | |  |  | | --- | --- | | Rezeptur |  | | Selbstdispensation |  | | Mischform |  | | |  |
|  |  | |  |
|  | |  |  | | --- | --- | | Stammkundschaft |  | | Passantenkundschaft |  | | in etwa ausgeglichen |  | | |  |
|  |  | |  |
| **4.4** | **Name und Ort der Apotheke / Datenschutz** | |  |
|  | Dieser Fragebogen wird in anonymer Form ausgewertet. Die Adressangaben  dienen lediglich zur statistischen Überprüfung um zu verhindern, dass wir Sie nach dem Zurücksenden dieses Fragebogens versehentlich nochmals mit  einem Reminder kontaktieren. Die Adressangaben werden von den Antworten  getrennt und anschliessend durch mich vernichtet, damit keine Zuteilung der  Antworten zu den einzelnen Apotheken mehr möglich wird. | |  |
|  | Name der Apotheke: | |  |
|  | Ort der Apotheke: | |  |
|  |  | |  |
|  |  | |  |
|  |  | |  |

**Vielen Dank!!**
